# Synergistic activity of colistin and stilbenoids against *Pseudomonas aeruginosa* clinical isolates

**DOI:** 10.1128/spectrum.01534-26

**Published:** 2026-09-22

**Authors:** Nicola Mangieri, Francesca Annunziata, Elio Rossi, Valerio Baldelli, Andrea Pinto, Sabrina Dallavalle, Diego Mora, Stefania Arioli

**Affiliations:** 1 Department of Food, Environmental and Nutritional Sciences (DeFENS), University of Milan 9304, Milan, Italy; 2 Department of Biosciences, University of Milan 9304, Milan, Italy; University of Manitoba, Winnipeg, Manitoba, Canada

**Keywords:** colistin, synergy, cystic fibrosis, stilbenoids, *Pseudomonas aeruginosa*

## Abstract

*Pseudomonas aeruginosa* is a major opportunistic pathogen responsible for chronic lung infections in cystic fibrosis (CF) patients, contributing significantly to morbidity and mortality. In this study, we investigated the synergistic activity of colistin combined with two natural stilbenoids, pterostilbene and (±)-*trans*-ε-viniferin, against clinical *P. aeruginosa* isolates from CF patients. Synergy was assessed through minimum inhibitory concentration (MIC) determination and time–kill assays. The combination of colistin with pterostilbene reduced colistin MIC values in all tested strains, with the strongest effect observed in a colistin-resistant isolate (eightfold reduction, fractional inhibitory concentration index = 0.188). Time–kill assays confirmed enhanced bactericidal activity, with faster and more effective bacterial killing compared to colistin alone. According to the known mechanism of action of colistin, medium supplementation with purified lipopolysaccharide (LPS) reduced colistin activity on *P. aeruginosa*. Medium supplementation with stilbenoids partially restored colistin activity, reducing the negative effect of LPS. Flow cytometry analyses showed increased membrane permeability in the presence of the combination of colistin and pterostilbene, whereas elevated concentrations of divalent cations reduced this effect, highlighting the role of electrostatic interactions in colistin/pterostilbene membrane damage. Overall, these findings suggest that pterostilbene acts as a colistin adjuvant *in vitro*, enhancing its antimicrobial activity against *P. aeruginosa* through a mechanism involving cell membrane damage.

**IMPORTANCE:**

*Pseudomonas aeruginosa* is a major cause of chronic lung infections in cystic fibrosis patients and is increasingly difficult to treat because of antibiotic resistance. Colistin remains one of the few effective last-line therapies against this bacterial pathogen, but its clinical use is limited by severe dose-dependent toxicity. In this study, we show that pterostilbene, a natural stilbenoid compound, enhances *in vitro* the activity of colistin against clinical *P. aeruginosa* isolates. The combination improved colistin efficacy, increased membrane damage, and partially overcame lipopolysaccharide-mediated reduction of colistin activity. Importantly, these effects were achieved using low concentrations of pterostilbene under the experimental conditions tested.

## INTRODUCTION

*Pseudomonas aeruginosa* is a ubiquitous gram-negative bacterium widely distributed in soil and aquatic environments. It is an opportunistic pathogen capable of causing a broad range of infections, particularly in immunocompromised individuals (1). This bacterium is frequently isolated in hospital settings, and it is associated with infections in vulnerable patients, including those with burns, ventilator-associated pneumonia, and bloodstream infections (2). *P. aeruginosa* establishes chronic colonization in the lungs of patients with cystic fibrosis (CF), where it represents the dominant pathogen that causes persistent respiratory infections and significantly contributes to morbidity and mortality (3). CF is a severe inherited disorder caused by mutations in the gene encoding the CF transmembrane conductance regulator (CFTR). Dysfunctional CFTR leads to impaired chloride transport and ionic imbalance in the airway epithelium, resulting in dehydration of the airway surface liquid and thick, viscous mucus. These alterations create a pulmonary environment that is particularly favorable for *P. aeruginosa* colonization and persistence (4). In CF patients, chronic *P. aeruginosa* infection persists despite prolonged and aggressive antibiotic treatments and is associated with a progressive decline in lung function and increased mortality. The management of chronic *P. aeruginosa* infections in CF patients remains a major clinical challenge and a significant global healthcare concern (5).

Considering the widespread emergence of antimicrobial resistance and the time-consuming, resource-intensive process required for the development of new antibiotics, alternative strategies are urgently needed to preserve the efficacy of existing therapies against multidrug-resistant (MDR) bacterial pathogens and to limit their side effects. One promising approach involves identifying compounds that can enhance the activity of currently available antibiotics, including natural or synthetic antimicrobial agents used in combination therapies. Although colistin, also known as polymyxin E, remains one of the most effective last-line antibiotics against gram-negative bacteria and resistance to it is still relatively limited (5), its clinical use is constrained by significant nephrotoxicity and neurotoxicity (6). The incidence of colistin-induced nephrotoxicity has been reported to range from 18% to 43%, particularly in patients receiving high-dose regimens or concomitant nephrotoxic agents (7). Cannatelli et al. (8) investigated the combination of colistin and resveratrol, a stilbenoid belonging to a class of polyphenolic compounds primarily studied for their health-promoting properties, against a collection of MDR gram-negative pathogens (8). While resveratrol alone lacked antimicrobial activity, it significantly potentiated colistin activity when used in combination. Similarly, Wang et al. (9) reported a marked synergistic effect of the colistin/resveratrol combination against *P. aeruginosa* isolates and their biofilms, both *in vitro* and *in vivo*, even though resveratrol alone exhibited no intrinsic antibacterial activity (9). Beyond antimicrobial potentiation, resveratrol has also shown the mitigation of colistin-induced nephrotoxicity through Nrf2-mediated antioxidant responses and inhibition of ferroptosis in murine models and renal epithelial cells (10). Additionally, grape-derived compounds such as proanthocyanidin extract significantly reduced colistin-induced nephrotoxicity by attenuating oxidative damage and caspase-mediated apoptosis (6). Other stilbenoids have also demonstrated synergistic effects with polymyxins. For instance, Zhou et al. (11) demonstrated that pterostilbene potentiates polymyxin B activity against mobile colistin resistance-positive *Escherichia coli* strains, although neither compound exhibited significant activity when used alone (11).

**Fig 1:**
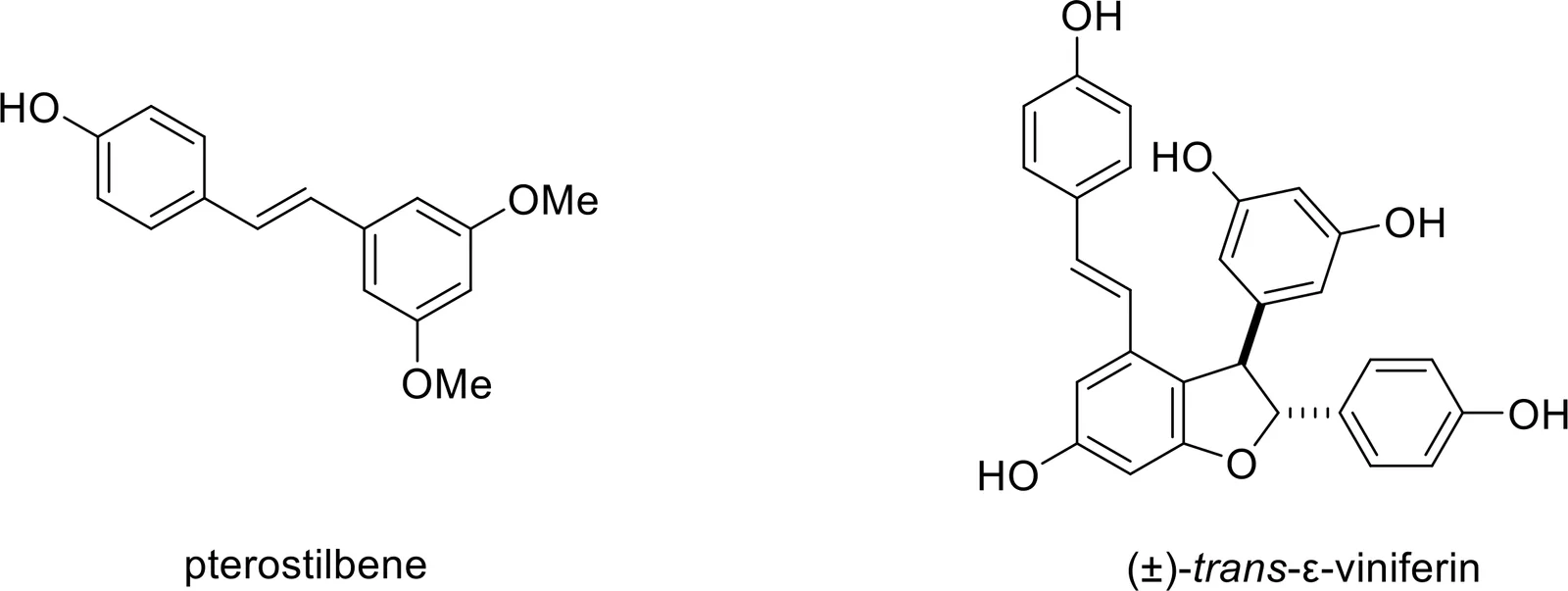
Structure of stilbenoids.

In the present study, we investigated the synergistic activity of colistin in combination with (i) pterostilbene, a monomeric stilbenoid produced in certain plant species and accumulated in response to biotic and abiotic stress, and (ii) (±)-*trans*-ε-viniferin, a resveratrol dimer commonly produced in *Vitis vinifera* as a stress-induced defense-related metabolite (Fig. 1). These combinations were evaluated against six clinical isolates of *P. aeruginosa* from CF patients. Synergistic effects were evaluated *in vitro* through minimum inhibitory concentration (MIC) determination and time–kill assays. Furthermore, we explored the potential mechanism underlying this interaction by assessing membrane damage induced by the combined treatment.

## MATERIALS AND METHODS

### Chemical synthesis

Pterostilbene, reagents, and solvents were purchased from commercial suppliers and used without further purification. The ^1^H NMR and ^13^C NMR spectra were recorded with a Bruker Avance Neo 400 MHz spectrometer. Chemical shifts (*δ*) are expressed in ppm, and coupling constants (*J*) are in hertz. Thin-layer chromatography (TLC) was performed using commercial silica gel 60 F254 aluminum sheets and RP-TLC plates (Merck). Compounds on TLC plates were detected under UV light at 254 and 365 nm or were revealed by spraying with 10% phosphomolybdic acid in ethanol. (±)-*Trans*-ε-viniferin was synthesized following the procedure reported by Mattio et al. (12). Briefly, to a solution of resveratrol (1 eq) in MeOH (20 mL), a solution of FeCl_3_·6H_2_O (1.01 eq) in water (20 mL) was added dropwise. The mixture was stirred at room temperature for 48 h. MeOH was evaporated, and the residue was diluted with water (30 mL) and extracted with EtOAc (4 × 50 mL). The combined organic layers were dried over anhydrous Na_2_SO_4_ and concentrated under reduced pressure. The residue was purified by FC (cyclohexane/acetone 3:2), and a mixture of (±)-*trans*-ε-viniferin and *cis-*analog was obtained. Further purification by reverse-phase column chromatography (H_2_O/MeOH 2:3) gave the final compound (yield 15%) as a light green amorphous solid: *R_f_*: 0.30 (cyclohexane/acetone 3:2) or 0.42 (H_2_O/MeOH 2:3); ^1^H NMR (300 MHz, methanol-*d*_4_): δ: 7.17–7.10 (m, 2H), 7.07–7.01 (m, 2H), 6.82 (d, *J* = 16.2 Hz, 1H), 6.78–6.73 (m, 2H), 6.67–6.62 (m, 2H), 6.63 (s, 1H), 6.56 (d, *J* = 16.2 Hz, 1H), 6.24 (d, *J* = 2.1 Hz, 1H), 6.20–6.14 (m, 3H), 5.36 (d, *J* = 6.6 Hz, 1H), and 4.34 (d, *J* = 6.6 Hz, 1H); ^13^C NMR (100 MHz, methanol-*d*_4_): δ: 162.8, 160.1, 159.8, 159.7, 158.5, 158.4, 147.4, 137.0, 136.9, 133.9, 130.5, 130.4, 128.8, 128.2, 123.8, 123.7, 120.1, 116.4, 116.3, 107.5, 104.4, 102.2, 96.9, 94.9, and 58.3.

The identity of (±)-*trans*-ε-viniferin was confirmed by ^1^H NMR spectroscopy. The corresponding spectrum is provided in Fig. S1. The purity (>95%) of the compound was confirmed by HPLC analysis (Fig. S2). (±)-*Trans*-ε-viniferin was stored in dry form at −20°C and dissolved in DMSO at the desired final concentration prior to use in biological assays.

### Cultivation conditions

Six clinical isolates of *P. aeruginosa* from chronically infected CF patients were present in our collection, previously isolated and characterized (13). *P. aeruginosa* DSM 1117 was purchased from Leibniz culture collection (DSMZ). The bacterial strains were routinely cultivated in LB broth (tryptone 10 g/L, NaCl 5 g/L, and yeast extract 5 g/L) at 37°C. The strains were stored at −80°C in LB supplemented with 20% (vol/vol) glycerol.

### MIC determination and time–kill assay

MIC and time–kill assays were performed in cation-adjusted Mueller–Hinton (MH) broth (Oxoid, Basingstoke, UK) except for PACF 177.1, which was tested in LB due to its inability to grow properly in MH. MIC was determined using the broth microdilution technique in accordance with EUCAST recommendations (14). Colistin sulfate salt was purchased from Sigma-Aldrich (Sigma code C4461), resuspended in water, and filter-sterilized using a 0.22 µm syringe filter. The tested concentrations ranged from 0.125 to 128 μg/mL. The same concentration range was applied to pterostilbene and (±)-*trans*-ε-viniferin (both dissolved in DMSO). To determine the initial bacterial inoculum, the total cell count and viability of microbial cultures were assessed by flow cytometry. The bacteria were grown in LB broth at 37°C for 24–48 h and subsequently diluted in filtered phosphate-buffered saline (PBS, pH 7.4). An event rate below 3,000 events/s was maintained during acquisition. Samples were stained with 0.1 µM SYTO 24 (Thermo Fisher Scientific) and 0.2 µM propidium iodide (PI, Sigma-Aldrich) and incubated in the dark at 37°C for 15 min. Flow cytometric analysis was performed using a BD Accuri C6 Plus flow cytometer (BD Biosciences, Milan, Italy) with threshold settings of FSC-H 3000 and SSC-H 1000. All parameters were collected using a logarithmic signal. Green fluorescence (SYTO 24) was detected in the FL1 channel (excitation 488 nm, emission 530/30 nm), while red fluorescence (PI) was detected in the FL3 channel (excitation 488 nm, emission 670 nm LP). Electronic gating on SYTO 24/PI density plots was used to determine the cell density (events/mL). The bacterial suspensions for MIC assay were diluted to a final cell density of 5 × 10^5^ SYTO^+^/mL. The final volume of each well was 0.2 mL. Each plate contained eight control wells with bacterial inoculum without antimicrobial agents and eight wells without bacterial inoculum. Microplates were incubated at 37°C for 24–48 h. Experiments were conducted in duplicate. The MIC was defined as the lowest concentration of the antimicrobial agent that completely inhibited visible bacterial growth. The fractional inhibitory concentration index (FICI) was calculated as the sum of the fractional inhibitory concentrations of each compound, according to the formula FICI = (MIC_A in combination / MIC_A alone) + (MIC_B in combination / MIC_B alone), and synergy was defined as FICI ≤0.5 (15). For compounds with MIC values exceeding the highest concentration tested (>128 µg/mL), a conservative value of 256 μg/mL was used for FICI calculations. Based on MIC values, four different multiples of the colistin MIC were evaluated, namely, 4× MIC, 1× MIC, 0.5× MIC, and 0.25× MIC, which were tested alone and in combination with pterostilbene or (±)-*trans-*ε-viniferin at a final concentration of 16 µg/mL. The inoculum of *P. aeruginosa* strains was standardized by flow cytometry as described above to a final cell density of 5 × 10⁵ SYTO24^+^/mL. Samples were collected at 0, 3, 6, and 24 h, serially diluted in PBS, and plated on Plate Count Agar (Scharlab, Barcelona, Spain). Plates were incubated at 37°C for 24 h.

### Role of lipopolysaccharide in colistin activity

Lipopolysaccharide (LPS) from *P. aeruginosa* ATCC 10145 was purchased from Sigma-Aldrich (Sigma code L9143). LPS was resuspended in water and filter-sterilized using a 0.22 µm syringe filter. MIC assays in the presence of LPS were performed as described above using *P. aeruginosa* DSM 1117. Minimum bactericidal concentration (MBC) was determined by spotting 10 µL from each well of the MIC plates onto Plate Count Agar (Scharlab) after 24 h of incubation. Petri dishes were incubated at 37°C for 24 h, and bacterial growth was assessed by the presence of at least one colony on agar medium. The MBC was effectively deemed to be the minimum concentration of antimicrobial capable of inactivating more than 99.9% of the bacteria present (16).

### Role of stilbenoids in cell membrane integrity

To determine whether the combination of colistin and pterostilbene increases cell membrane permeability, PI fluorescence was monitored by flow cytometry after staining cells with SYTO24 and PI. SYTO24 permeates the membrane of total cells regardless of cell membrane damage and stains nucleic acids with green fluorescence, while PI penetrates only bacteria with damaged membranes, causing a reduction in SYTO24 fluorescence when both dyes are present (12). The strain PACF 177.1 was cultivated in LB broth for 24 h. Subsequently, the cell suspension was divided into different conditions. Colistin was maintained at a fixed concentration of 64 μg/mL. Pterostilbene was used at 0, 2, 4, 8, and 16 μg/mL in the presence or absence of colistin (64 μg/mL). The exposure time of bacterial cells to the antimicrobials was set at 3 h. The same experiment, with slight modifications, was performed in the presence of different concentrations of divalent cations (Mg²^+^ and Ca²^+^). After cultivation of strain PACF 177.1 in LB for 24 h, the culture was diluted 1:20 in MH broth (final volume: 1 mL). Samples were tested in the presence of 16 μg/mL pterostilbene, 64 μg/mL colistin, and their combination at the same concentrations. Each condition was evaluated under three different cation concentrations: (i) 3 mg/L Ca²^+^ and 5.8 mg/L Mg²^+^, (ii) 23 mg/L Ca²^+^ and 11 mg/L Mg²^+^, and (iii) 230 mg/L Ca²^+^ and 110 mg/L Mg²^+^. After 3 h of exposure, the results were acquired by flow cytometry as previously described. The assays were conducted in triplicate.

## RESULTS

### MICs of colistin, pterostilbene, and their combination

Bacterial susceptibility to the tested antimicrobials is reported in Table 1, whereas the effect of the solvent (DMSO) is shown in the Table S1. In six out of seven cases, the MICs of pterostilbene and (±)-*trans*-ε-viniferin were higher than the maximum concentration tested, and in one case were equal to the maximum tested concentration (128 μg/mL). In six out of seven strains, the MIC values for colistin ranged from 0.5 to 1 μg/mL.

**TABLE 1:** MIC results reported in microgram per milliliter

Strains of *P. aeruginosa*	Colistin	Pterostilbene	Colistin + pterostilbene^*a*^	FICI^*b*^	(±)-*Trans*-ε-viniferin	Colistin + (±)-*trans*-ε- viniferin^*a*^	FICI^*b*^
PACF 2014	1	>128	0.5	0.562	>128	1	1.062
PACF 134	1	128	0.5	0.625	128	1	1.125
PACF 124.1	1	>128	0.25	**0.312**	>128	0.5	0.562
PACF 124.2	0.5	128	0.125	**0.375**	128	0.25	0.625
PACF 177.1	64	>128	8	**0.188**	>128	64	1.062
PACF 30.1	0.5	>128	<0.125	**0.312**	>128	0.125	**0.312**
DSM 1117	0.5	>128	0.125	**0.312**	>128	0.25	0.562

^*a*^ 16 μg/mL.

^*b*^ FICI, fractional inhibitory concentration index. In bold are the reported values that showed synergy (FICI ≤0.5).

The addition of pterostilbene (16 μg/mL) decreased the colistin MIC by at least twofold in all isolates, with a maximum decrease of eightfold. Synergy (FICI ≤0.5) between pterostilbene and colistin was observed in four out of six *P. aeruginosa* CF isolates (Table 1). Remarkably, the greatest reduction, with a FICI of 0.188, was observed in the isolate PACF 177.1, classified as colistin resistant according to EUCAST clinical breakpoints (v16.0) for *Pseudomonas* spp. (17), due to the synergy between colistin and pterostilbene.

The dimer (±)-*trans*-ε-viniferin decreased colistin MIC values in three out of six cases, with reductions up to fourfold when combined with colistin. In one out of six cases (PACF 30.1), it showed synergy (FICI ≤0.5) (Table 1).

### Genomic basis of colistin resistance in *P. aeruginosa*

The whole genomes of the *P. aeruginosa* isolates were sequenced. No plasmids associated with antibiotic resistance were detected in any of the strains, including the colistin-resistant PACF 177.1. Furthermore, no acquired colistin resistance determinants were identified in PACF 177.1.

Colistin resistance in *P. aeruginosa* is primarily mediated by chromosomal mutations that lead to LPS modification, particularly through the addition of 4-amino-4-deoxy-L-arabinose (L-Ara4N) to the phosphate groups of lipid A and the core oligosaccharide, thereby reducing the net negative charge of the bacterial outer membrane (18). These modifications decrease the electrostatic interaction between colistin and LPS, resulting in reduced antibiotic binding and increased resistance. The regulation of LPS modification is multifactorial and largely controlled by mutations affecting the two-component regulatory systems PmrAB and PhoPQ. In PACF 177.1, we identified two mutations (Table 2) in the *pmrB* gene (Ala211Val and Tyr345His), both previously reported to be associated with colistin resistance (18). These mutations are located within functional domains of PmrB and are known to promote constitutive activation of the PmrAB regulatory system. Such activation leads to upregulation of the *arn* operon, which encodes enzymes responsible for L-Ara4N biosynthesis and its incorporation into lipid A, ultimately conferring resistance. In addition, we detected an in-frame deletion in the *phoQ* gene (Leu273_Gly274del), previously described in colistin-resistant isolates (19). Alterations within the sensor domain of PhoQ can result in dysregulated activation of the PhoPQ system, further enhancing the expression of genes involved in LPS remodeling. The combined presence of activating mutations in *pmrB* and structural alterations in *phoQ* likely contributes to enhanced, stable LPS modification, thereby explaining the observed colistin-resistant phenotype in PACF 177.1 despite the absence of plasmid-mediated resistance mechanisms.

**TABLE 2:** Genomic mutations associated with colistin resistance reported in *P. aeruginosa* PACF 177.1

Related genes	Type of mutation	References
*pmrB*	Ala211Val	(18)
*pmrB*	Tyr345His	(18)
*phoQ*	Leu273_Gly274del (in-frame deletion)	(19)

### Effect of exogenous LPS and stilbenoids on colistin susceptibility in *P. aeruginosa* DSM 1117

The role of exogenous LPS from *P. aeruginosa* was investigated by determining the colistin MIC and MBC on *P. aeruginosa* DSM 1117. In the presence of exogenous LPS, the colistin MIC increased from 0.5 to 16 μg/mL. The addition of stilbenoids to MH broth resulted in intermediate values, with the colistin MIC decreasing from 16 to 4 μg/mL for both molecules (Table 3). The MBC values followed the same trend. These findings suggest that LPS may sequester colistin, reducing its activity, while the stilbenoids partially restore susceptibility when combined with colistin.

**TABLE 3:** Results of MIC and MBC on *P. aeruginosa* DSM 1117

Antimicrobial combinations	MIC (μg/mL)	MBC (μg/mL)
Colistin	0.5	0.5
Colistin + LPS^*a*^	16	16
Colistin + LPS^*a*^ + pterostilbene^*b*^	4	4
Colistin + LPS^*a*^ + (±)-*trans*-ε-viniferin^*b*^	4	4

^*a*^ The LPS concentration was 50 μg/mL.

^*b*^ The stilbenoid concentration was 16 μg/mL.

In a further assay aimed at evaluating the LPS concentrations capable of altering the MIC of colistin, the concentration of the antibiotic was kept constant (0.5 μg/mL) in all wells at its MIC value. The LPS concentrations tested ranged from 0.1 to 50 μg/mL. The highest concentrations of LPS (25 and 50 μg/mL) were able to increase the MIC. At 12.5 μg/mL and all lower concentrations tested, LPS did not affect the colistin MIC and MBC of *P. aeruginosa* DSM 1117.

### Time–kill assay

**Fig 2:**
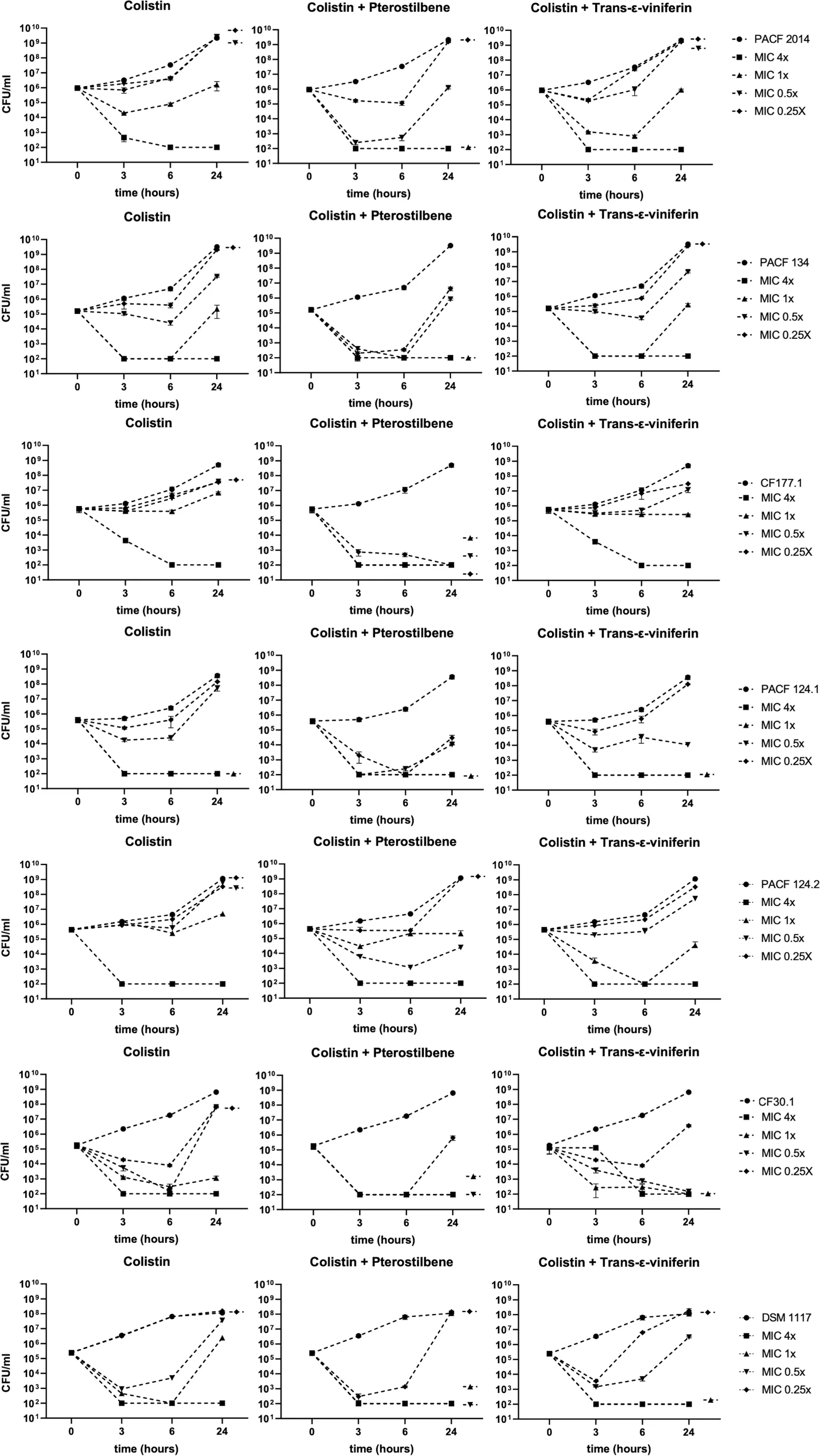
Time–kill curves of *P. aeruginosa* strains treated with colistin alone or in combination with stilbenoids. Bacterial cultivability (CFU/mL) was monitored over 24 h at different multiples of the MIC (4×, 1×, 0.5×, and 0.25× MIC). Pterostilbene and (±)*-trans*-ε-viniferin were tested at a fixed concentration of 16 μg/mL. Data are presented as the means ± standard deviations of two independent biological replicates.

To investigate the combination of colistin with stilbenoids, time–kill assays were performed based on the previously determined MIC values. The experiment was conducted using four different multiples of the MIC, along with a control without antibiotic addition and with the stilbenoid solvent (DMSO) ( Fig. S3). As shown in Fig. 2, in the absence of colistin exposure, starting from 5 × 10^5^ CFU/mL as inoculum, all strains reached at least 10^8^ CFU/mL in 24 h. By defining bactericidal activity as a ≥99.9% reduction in CFU per milliliter (16), in all *P. aeruginosa* strains tested, colistin at 4× MIC was bactericidal within a maximum of 6 h of exposure (Fig. 2). In the presence of pterostilbene, colistin at 4× MIC resulted in complete bacterial killing within 3 h in all cases. Except for *P. aeruginosa* PACF 124.2, the combination of pterostilbene with colistin at 1× MIC showed a bactericidal effect within 24 h. In all strains tested, pterostilbene enhanced the efficacy of colistin alone. Notably, the synergistic effect between colistin and pterostilbene was particularly evident in *P. aeruginosa* PACF 177.1, which was classified as colistin resistant, with bactericidal activity observed at all concentrations tested, whereas colistin alone was effective only at 4× MIC. Regarding (±)-*trans*-ε-viniferin, in most strains, it enhanced its antimicrobial activity; however, consistent with the MIC results, pterostilbene exhibited a stronger bactericidal effect across the different isolates tested, both in terms of exposure time and the amount of colistin required to achieve a bactericidal effect.

### Dose-dependent increase in membrane damage induced by pterostilbene in combination with colistin

**Fig 3:**
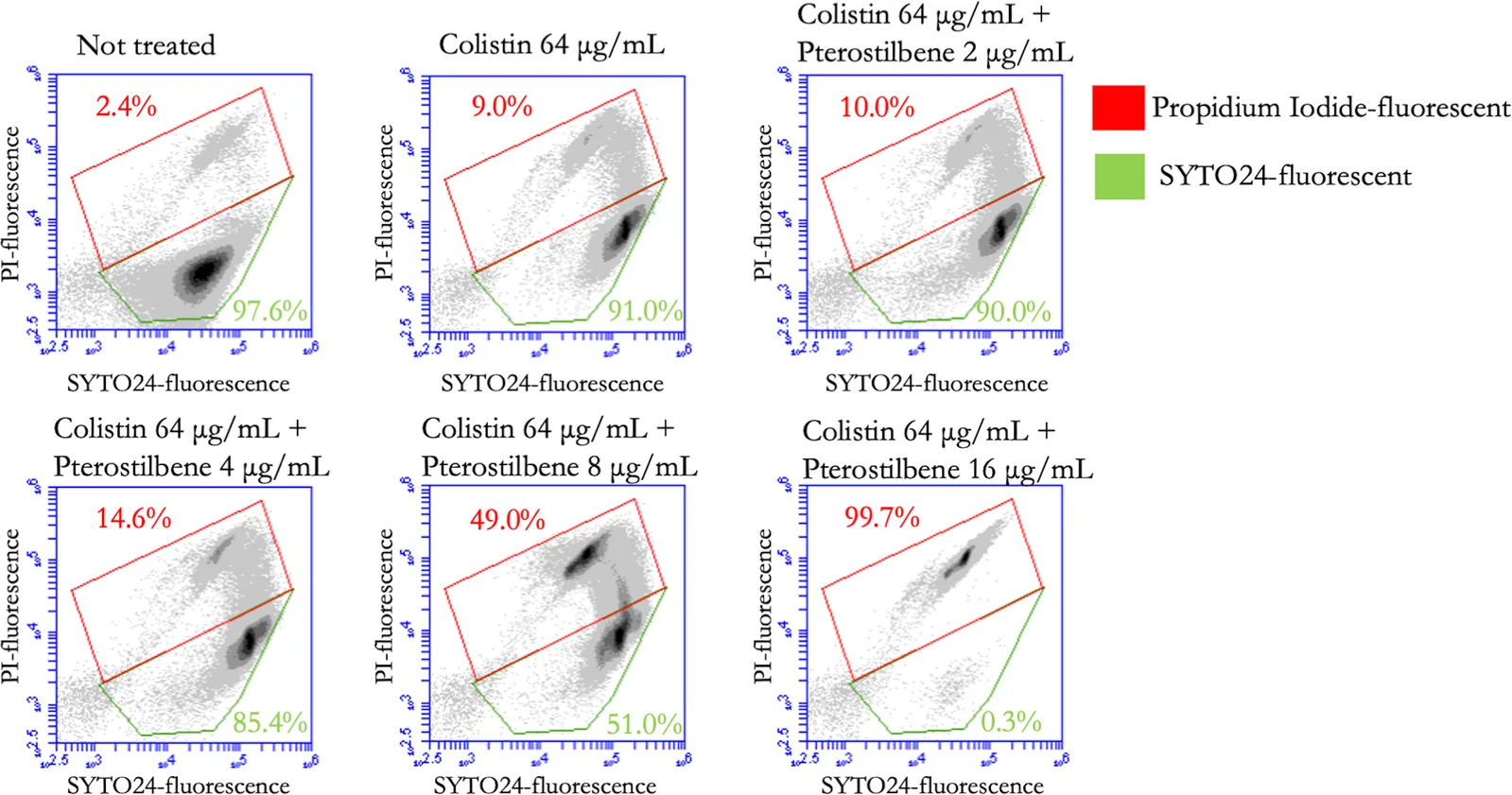
Flow cytometry analysis of membrane permeability in *P. aeruginosa* PACF 177.1. Representative dot plots from one of the replicates showing SYTO 24 (green fluorescence) and propidium iodide (PI, red fluorescence) staining after 3 h of exposure to colistin (64 μg/mL) and increasing concentrations of pterostilbene (0–16 μg/mL).

The colistin-resistant *P. aeruginosa* PACF 177.1 was employed to assess whether the observed synergistic effect was associated with increased membrane permeability. Membrane integrity was assessed by flow cytometry using SYTO 24 and PI staining. As shown in the representative dot plots (Fig. 3), untreated cells exhibited an intact membrane (97.4% SYTO 24 fluorescent). Exposure to colistin at its MIC value (64 μg/mL) resulted in a modest increase in PI-positive events, reflecting limited cell membrane damage.

**Fig 4:**
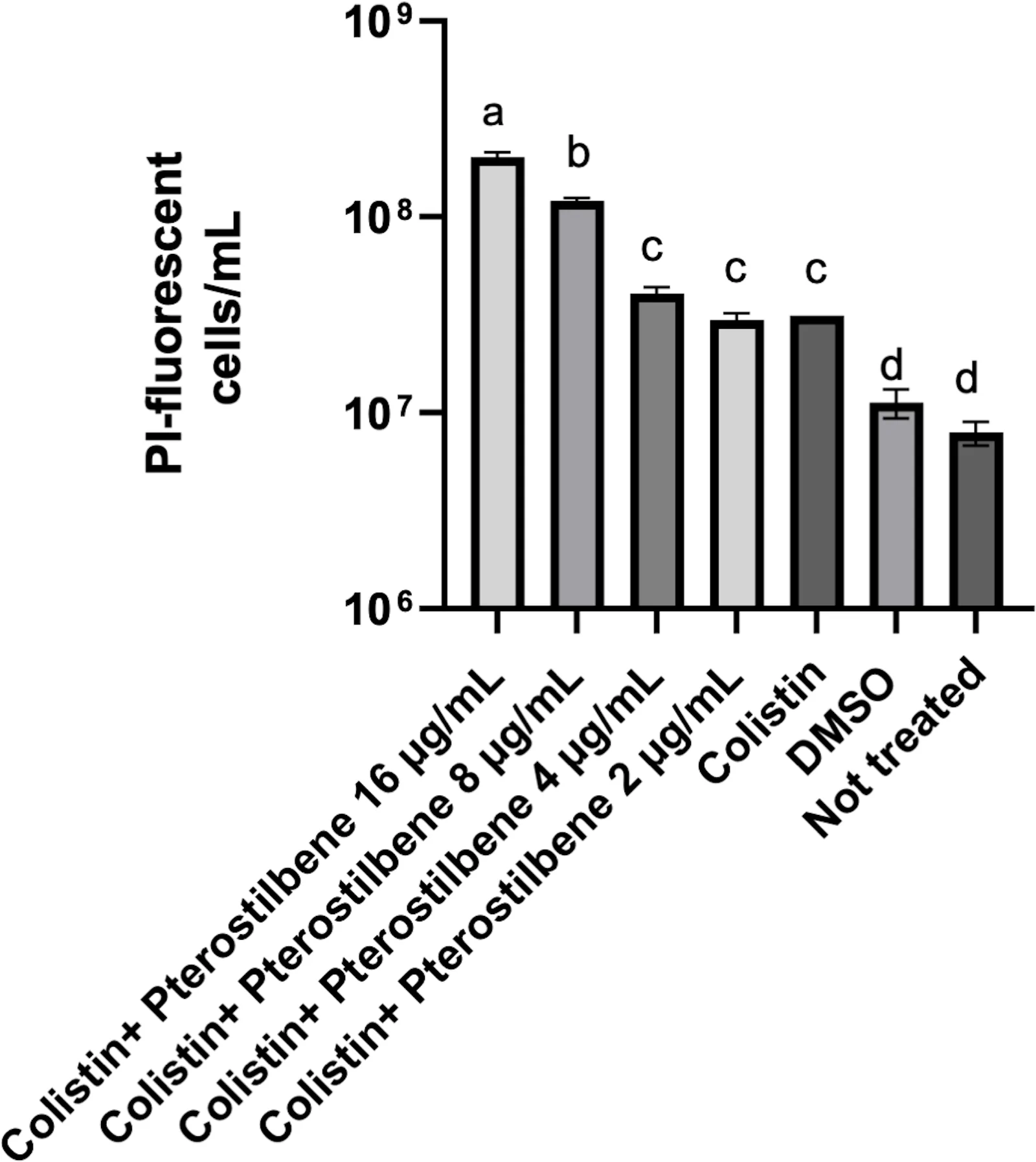
Effect of pterostilbene on colistin-induced cell membrane permeabilization in *P. aeruginosa* PACF 177.1. The number of propidium iodide-fluorescent events (events/mL) was measured by flow cytometry after 3 h of exposure to colistin (64 μg/mL) in combination with increasing concentrations of pterostilbene (2–16 μg/mL). Colistin alone, DMSO (0.78% vol/vol), and untreated cells were included as controls. Data are presented on a logarithmic scale as means ± standard deviations. Different letters (a–d) indicate statistically significant differences between groups (*P* < 0.05) obtained by one-way ANOVA followed by Tukey’s test.

When colistin was combined with increasing concentrations of pterostilbene (2–16 μg/mL), a progressive shift in the cell population toward PI-positive events was observed, indicating a concentration-dependent increase in membrane permeabilization. At the highest concentration tested, nearly all cells were PI fluorescent, consistent with extensive membrane disruption. Quantitative analysis of PI-fluorescent events (Fig. 4) confirmed this trend, showing a significant increase in membrane permeability in the presence of the combination compared to colistin alone. Statistical analysis revealed a dose-dependent effect of pterostilbene, with higher concentrations leading to significantly greater numbers of PI-fluorescent cells (*P* < 0.05). These findings indicate that pterostilbene enhances colistin-mediated membrane damage, supporting a membrane-targeting mechanism.

**Fig 5:**
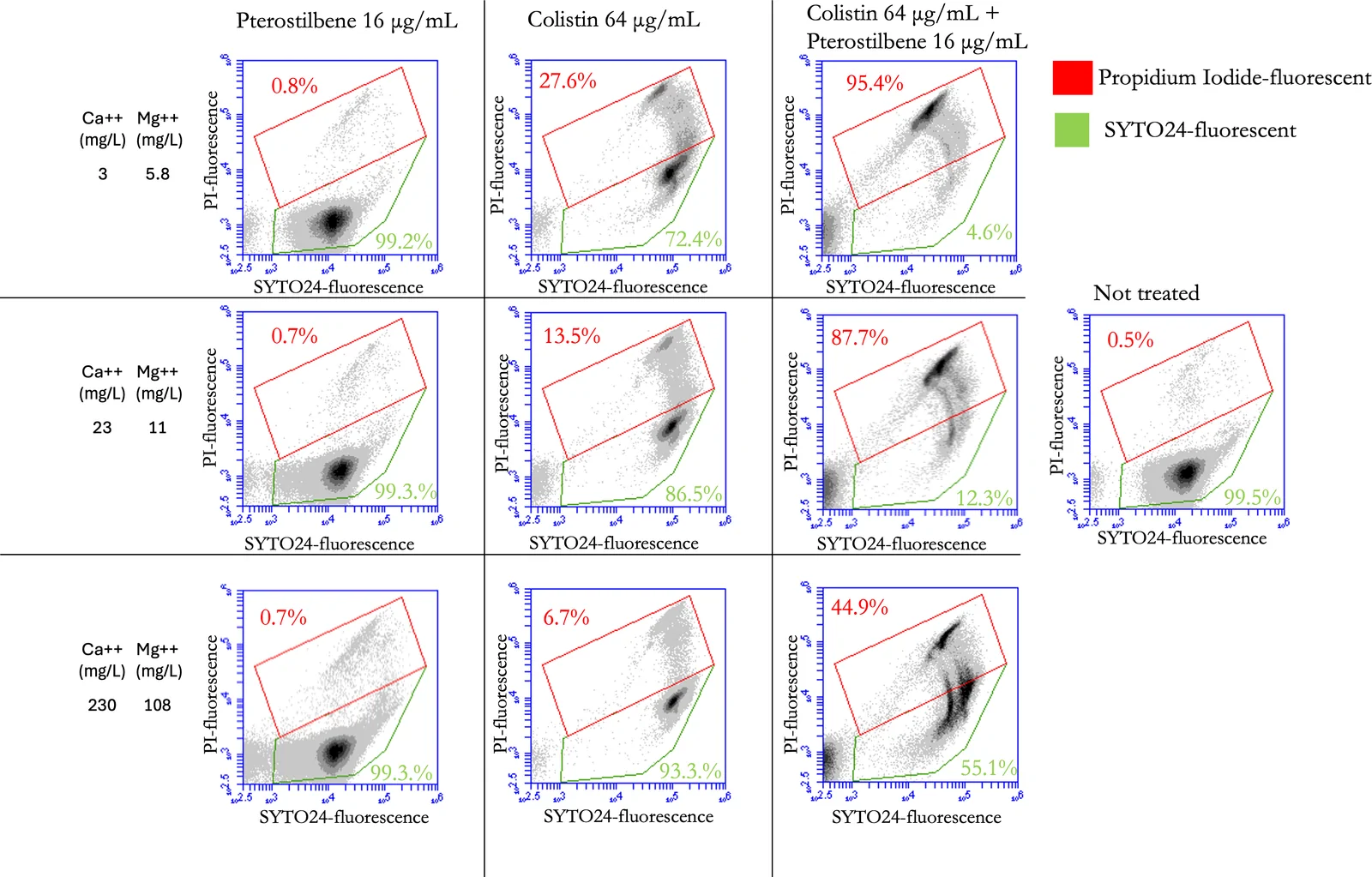
Effect of divalent cations on colistin-mediated membrane permeabilization. Representative dot plots are shown. Percentage of PI-positive cells in *P. aeruginosa* PACF 177.1 after 3 h of exposure to colistin (64 μg/mL), pterostilbene (16 μg/mL), and their combination under increasing concentrations of Ca²⁺ and Mg²⁺.

As shown in Fig. 5, the role of cations in enhancing colistin resistance is suggested. Treatment with colistin at 64 μg/mL showed a cation concentration-dependent effect, reducing the proportion of propidium iodide-fluorescent events from 27.6% at the lowest cation concentration to 6.7%. A similar but more pronounced trend was observed under the second condition, where the percentage decreased from 95.4% at low levels to 44.9% in the presence of excess cations. Pterostilbene showed no effect on membrane permeability at any of the cation concentrations tested. Two-way ANOVA revealed significant main effects for both treatment group [*F*(2, 9) = 67.59, *P* < 0.0001] and cation concentration level [*F*(2, 9) = 908.7, *P* < 0.0001], as well as a significant treatment × concentration interaction [*F*(4, 9) = 37.93, *P* < 0.0001], which accounted for 7.18% of the total variation. Post hoc Tukey’s multiple comparison test showed that the combined treatment (colistin + pterostilbene 16 μg/mL) resulted in significantly higher PI-positive cell counts than either pterostilbene (16 μg/mL) or colistin alone across all cation concentrations (all *P* < 0.001). In contrast, pterostilbene alone produced consistently low PI-positive cell counts irrespective of cation concentration (all *P* > 0.90), whereas colistin exhibited a concentration-dependent reduction, reaching values comparable to those of pterostilbene at the highest cation concentration (*P* = 0.2225).

## DISCUSSION

Chronic *P. aeruginosa* infection represents one of the main causes of morbidity and mortality in CF patients, largely due to its remarkable adaptive capacity and progressive acquisition of antibiotic resistance (1). Among last-line therapies, colistin remains an effective option; however, resistance and dose-dependent nephrotoxicity significantly limit its clinical use (5, 7). In this study, we demonstrate that pterostilbene acts as an effective antibiotic adjuvant, significantly enhancing colistin activity against *P. aeruginosa* clinical isolates from CF patients. The strongest synergy was observed in the colistin-resistant strain PACF 177.1, where the MIC decreased eightfold in the presence of pterostilbene (FICI = 0.188). Time–kill assays further confirmed the synergistic interaction. While colistin alone required 4× MIC to achieve bactericidal activity, the addition of pterostilbene accelerated bacterial killing and enabled bactericidal effects at lower concentrations. The synergistic activity of resveratrol, the most extensively studied stilbenoid, in combination with colistin has been previously shown to enhance antimicrobial efficacy against gram-negative bacteria (8). Stilbenoids were tested at a low concentration (16 μg/mL) to minimize cytotoxicity and non-specific stress responses, as well as to reduce potential interference from the solvent (DMSO), given their poor aqueous solubility. At this concentration, the final DMSO content was maintained below 1% (vol/vol) in the combination experiments. This sub-active dose was deliberately selected to evaluate modulatory and antibiotic-potentiating effects rather than direct antimicrobial activity while maintaining experimental conditions that are more plausibly translatable *in vivo*. The ability to achieve synergistic effects at lower adjuvant concentrations may support future investigations aimed at reducing the colistin dose required to achieve bactericidal activity. This observation is of particular interest in the context of CF, where patients are exposed to repeated and prolonged colistin therapy, with a consequent increased risk of dose-dependent nephrotoxicity (6, 7). However, whether such synergy can be translated *in vivo* will require dedicated pharmacokinetic, pharmacodynamic, and toxicity studies in appropriate animal models.

Polymyxins bind to lipid A through electrostatic interactions with negatively charged phosphate groups. This interaction promotes the organization of LPS into ordered hexagonal assemblies, forming rigid crystalline structures. These changes stiffen the outer membrane, leading to protrusions, membrane rupture, and leakage of cellular contents, ultimately allowing access to the inner membrane and causing bacterial death (20). Consistent with this mechanism, our supplementation with exogenous LPS further supports the central role of lipid A binding, as excess LPS reduced colistin efficacy by acting as a competitive target. In this context, colistin can bind not only to the LPS of the bacterial outer membrane but also to free LPS in the extracellular environment, thereby decreasing the amount of active antibiotic available to interact with bacterial cells. Both stilbenoids partially restored colistin activity in the presence of LPS (50 μg/mL), suggesting that they may interfere with LPS-mediated sequestration or alter membrane interactions.

Investigation of membrane permeability further supported a membrane-targeting mechanism. In PACF 177.1, the combination of colistin and pterostilbene induced a concentration-dependent increase in PI-positive cells, indicating progressive membrane disruption correlated with pterostilbene concentration. Since PI penetrates only cells with compromised membranes, these results suggest that pterostilbene enhances colistin-mediated membrane damage. Similar membrane-permeabilizing effects have been reported for stilbenes, including resveratrol derivatives, which can alter lipid bilayer organization and increase membrane permeability (9). The observed cation-dependent reduction in colistin activity further supports the central role of electrostatic interactions in polymyxin-mediated membrane disruption. Increasing concentrations of divalent cations markedly decreased the proportion of propidium iodide-fluorescent cells, indicating reduced membrane permeabilization. This effect is consistent with the well-established ability of cations such as Mg²^+^ and Ca²^+^ to stabilize the outer membrane by bridging adjacent LPS molecules and neutralizing the negative charges of lipid A phosphate groups (20, 21). By reinforcing these cation-mediated interactions, higher cation concentrations likely hinder the competitive displacement of divalent cations by colistin, thereby reducing its binding to LPS and impairing its bactericidal activity (22, 23).

Notably, pterostilbene did not restore colistin efficacy under these conditions, suggesting that its potentiating effect does not involve direct competition with cations for phosphate binding sites. Instead, it likely acts by modulating membrane organization. This effect may be related to the presence of methoxy groups, which increase the lipophilicity of pterostilbene and favor its partitioning into lipid environments (24), altering lipid packing or membrane dynamics and facilitating colistin interaction with LPS.

Pterostilbene has been reported to be pharmacologically safe, as no organ-specific or systemic toxicity was observed, even after intravenous administration at a high dose (30 mg/kg/day for 23 days), based on histopathological examination and routine hematological and clinical chemistry analyses (25). According to available experimental evidence, pterostilbene also exhibits potential health benefits in inflammatory dermatoses, photoprotection, cancer prevention and therapy, insulin sensitivity, glycemic control, lipid metabolism, cardiovascular diseases, aging, and cognitive function (26).

Although no plasmid-mediated colistin resistance determinants were identified in the isolates used, PACF 177.1 harbored mutations in *pmrB* (Ala211Val and Tyr345His) and an in-frame deletion in *phoQ* (Leu273_Gly274del), all previously associated with colistin resistance. These alterations likely promote constitutive activation of the PmrAB and PhoPQ regulatory systems, resulting in enhanced expression of genes involved in LPS remodeling, particularly the addition of L-Ara4N to lipid A. Such modifications reduce the net negative charge of the outer membrane, thereby decreasing colistin binding and explaining the resistant phenotype observed in this strain.

As previously reported, extracellular LPS released from *P. aeruginosa* can sequester colistin and significantly reduce its antimicrobial activity, with free LPS inhibiting colistin more effectively than membrane-bound LPS (27). Consistent with these findings, under our experimental conditions, the presence of free LPS reduced colistin efficacy in *P. aeruginosa* DSM 1117, increasing the colistin MIC from 0.5 to 16 μg/mL, whereas the presence of the tested stilbenoids partially restored colistin activity, lowering the MIC to 4 μg/mL. This observation suggests a possible mechanism by which extracellular LPS, released from dead or lysed bacterial cells, could act as a decoy target for colistin, thereby reducing its efficacy, which stilbenoids may partially restore. Taken together, our findings indicate that stilbenoids, particularly pterostilbene, can significantly potentiate colistin activity against CF-derived *P. aeruginosa*, including resistant isolates. Given the nephrotoxicity associated with high-dose colistin therapy (7), the identification of compounds capable of potentiating colistin activity remains an important research objective to improve the efficacy and safety profile of colistin-based therapies against gram-negative infections. However, this study has some limitations. The number of clinical isolates included was relatively limited, and the experiments were performed exclusively *in vitro*. Further pharmacokinetic, pharmacodynamic, and *in vivo* studies are required to determine the translational potential of this combination.

### Conclusion

Pterostilbene significantly enhanced colistin activity against *P. aeruginosa* isolates from cystic fibrosis patients, including a colistin-resistant strain harboring *pmrB* and *phoQ* mutations. The combination promoted membrane permeabilization and partially counteracted LPS-mediated resistance mechanisms. Notably, the potentiating effect was observed at low pterostilbene concentrations, supporting its potential as an efficient antibiotic adjuvant. These findings suggest that this strategy may enhance colistin efficacy while enabling dose reduction, potentially mitigating its dose-dependent toxicity. Further studies are warranted to assess the translational potential of this combination *in vivo*.

## Data Availability

The raw whole-genome sequencing data analyzed in this study are publicly available in the EMBL-EBI European Nucleotide Archive under BioProject accession number PRJEB24688.

## SUPPLEMENTAL MATERIAL

Supplemental figures and table spectrum.01534-26-s0001.pdf Fig. S1 to S3 and Table S1.

ASM does not own the copyrights to Supplemental Material that may be linked to, or accessed through, an article. The authors have granted ASM a non-exclusive, world-wide license to publish the Supplemental Material files. Please contact the corresponding author directly for reuse.
