# Supplementary material for: Synergistic activity of colistin and stilbenoids against Pseudomonas aeruginosa clinical isolates
Source: Microbiol Spectr. 2026 Sep 22;14(11):e01534-26. doi: 10.1128/spectrum.01534-26 (PMC13729214; doi:10.1128/spectrum.01534-26)

Supplementary Figure 1. ^{1}H NMR spectrum of (±)-*trans*-ε-viniferin.

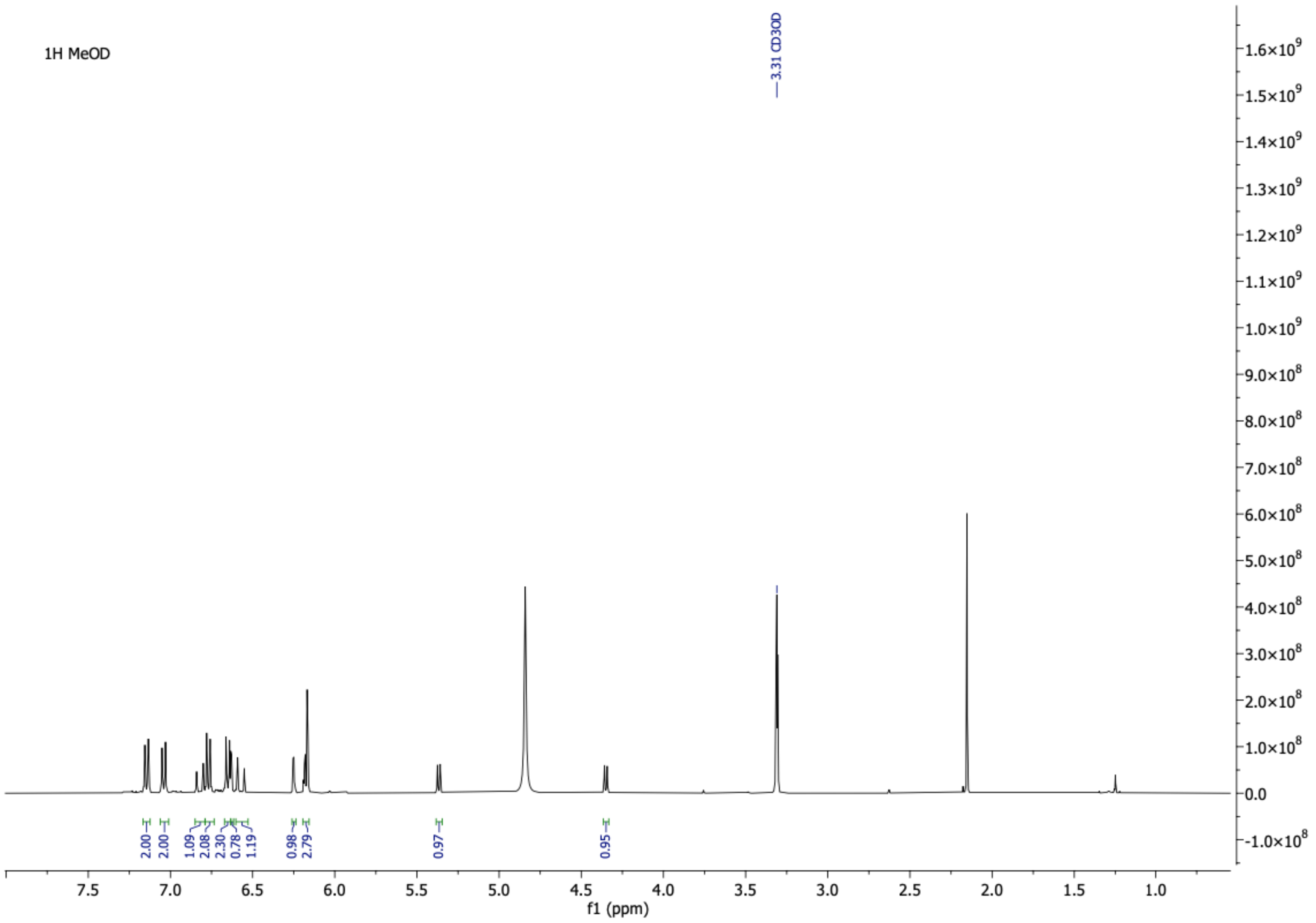

Supplementary Figure 2. Analytical HPLC chromatogram of (±)-*trans*-ε-viniferin. The chromatographic profile confirmed a purity of 96.42%, as determined by peak area normalization.

UNIMI

Project Name Pholiphenol

Reported by User: Breeze user (Breeze)

Breeze 2 HPLC System

SAMPLE INFORMATION

Sample Name:	epsilon vinif standard	Acquired By:	Breeze
Sample Type:	Unknown	Date Acquired:	2/26/2018 2:34:54 PM CET
Vial:	1	Acq. Method:	Polyphenol Zorbax 280nm
Injection #:	1	Date Processed:	1/13/2011 11:02:25 AM CET
Injection Volume:	20.00 ul	Channel Name:	W2489 ChA
Run Time:	60.00 Minutes	Sample Set Name	

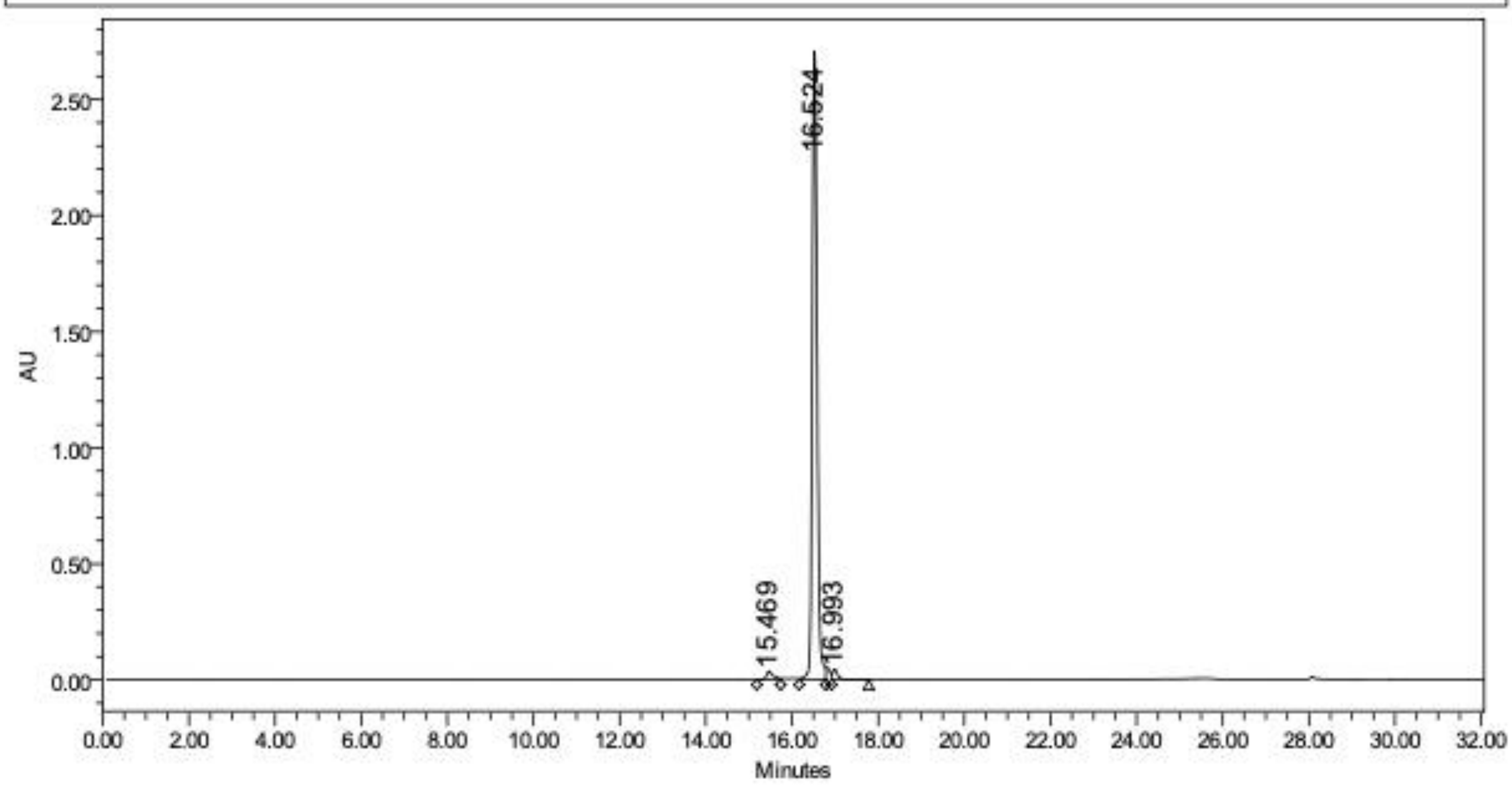

	RT (min)	Area (µV*sec)	% Area	Height (µV)	% Height
1	15.469	394635	1.82	35799	1.28
2	16.524	20948014	96.42	2722093	97.10
3	16.993	382845	1.76	45578	1.63

Supplementary Table 1. MIC and MBC results of DMSO reported in µg/mL. The concentration used for the combination assays was 8580 µg/mL (0.78% v/v).

Strains of *P. aeruginosa*	MIC	MBC
PACF 2014	137500	>137500
PACF 134	68750	137500
PACF 124.1	68750	137500
PACF 124.2	68750	137500
PACF 177.1	68750	137500
PACF 30.1	137500	>137500
DSM 1117	137500	>137500

Supplementary Figure 3. Time–kill curves of *P. aeruginosa* strains treated with DMSO at the same concentration (0.78% v/v; 8580 µg/mL) used in the combination assays. No significant differences in bacterial growth were observed between untreated controls and cultures exposed to DMSO.

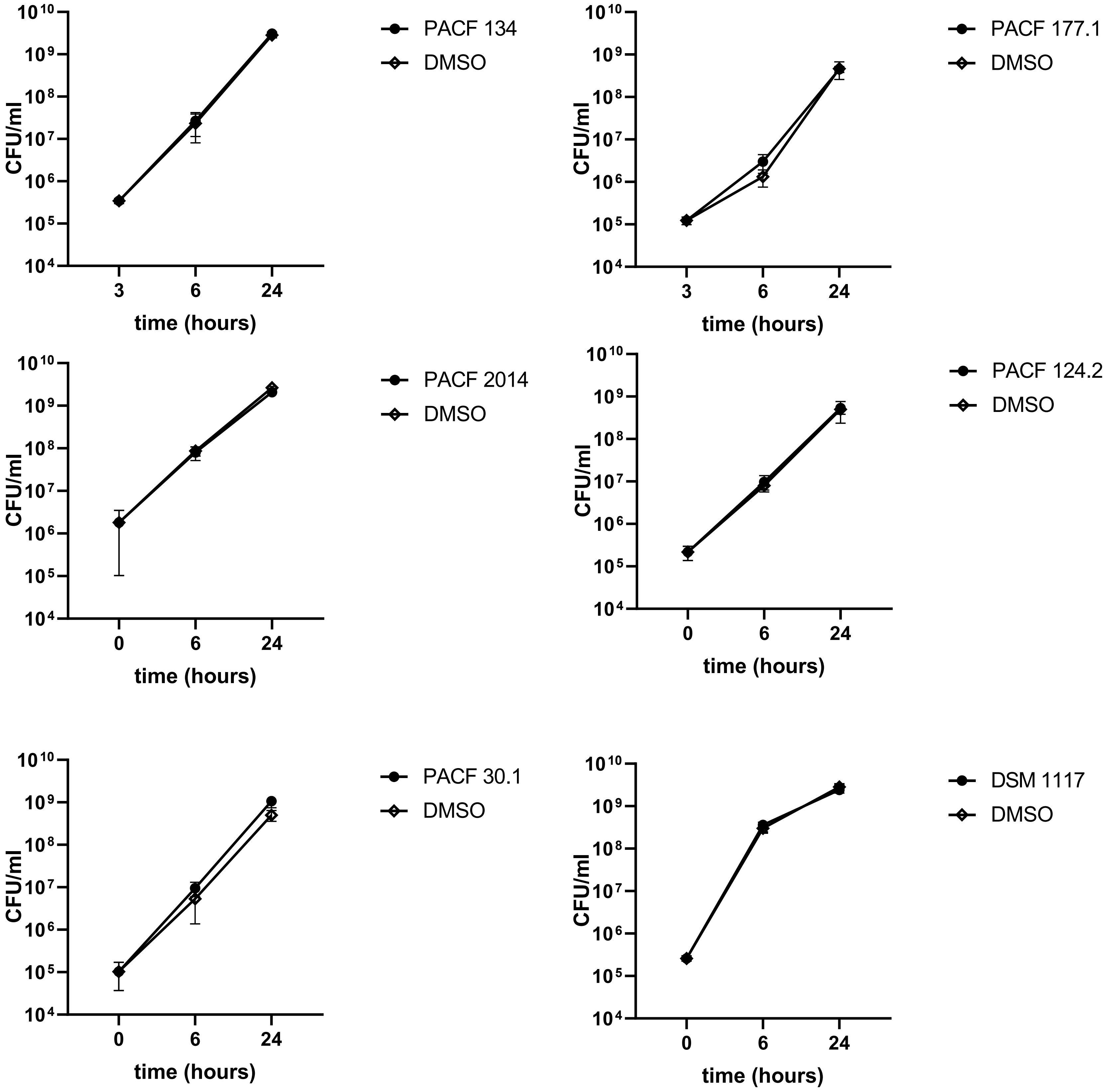

Supplement: Supplementary file 1 [file spectrum.01534-26-s0001.pdf]
